# Microfluidic Devices for HIV Diagnosis and Monitoring at Point-of-Care (POC) Settings

**DOI:** 10.3390/bios12110949

**Published:** 2022-11-01

**Authors:** Shebin Tharakan, Omair Faqah, Waseem Asghar, Azhar Ilyas

**Affiliations:** 1 Bio-Nanotechnology and Biomaterials (BNB) Lab, New York Institute of Technology, Old Westbury, NY 11568, USA; 2 Department of Biological & Chemical Sciences, New York Institute of Technology, Old Westbury, NY 11568, USA; 3 Department of Electrical Engineering and Computer Science, Florida Atlantic University, Boca Raton, FL 33431, USA; 4 Department of Electrical and Computer Engineering, New York Institute of Technology, Old Westbury, NY 11568, USA

**Keywords:** microfluidic, CD4+ T cells, miniaturization, detection assay, antibodies

## Abstract

Human immunodeficiency virus (HIV) is a global epidemic; however, many individuals are able to obtain treatment and manage their condition. Progression to acquired immunodeficiency syndrome (AIDS) occurs during late-stage HIV infection, which compromises the immune system, making it susceptible to infections. While there is no cure, antiretroviral therapy can be used provided that detection occurs, preferably during the early phase. However, the detection of HIV is expensive and resource-intensive when tested with conventional methods, such as flow cytometry, polymerase chain reaction (PCR), or enzyme-linked immunosorbent assays (ELISA). Improving disease detection in resource-constrained areas requires equipment that is affordable, portable, and can deliver rapid results. Microfluidic devices have transformed many benchtop techniques to on-chip detection for portable and rapid point-of-care (POC) testing. These devices are cost-effective, sensitive, and rapid and can be used in areas lacking resources. Moreover, their functionality can rival their benchtop counterparts, making them efficient for disease detection. In this review, we discuss the limitations of currently used conventional HIV diagnostic assays and provide an overview of potential microfluidic technologies that can improve HIV testing in POC settings.

## 1. Introduction

Human immunodeficiency virus or HIV is an RNA retrovirus that progresses into acquired immunodeficiency syndrome (AIDS) over a long period of time. The retrovirus attacks CD4+ T cells compromising the immune system in fighting infectious diseases [1]. HIV was first extracted from a patient in 1983 [2]. Since then, there are approximately 76 million individuals infected with HIV-1 worldwide. More antiretroviral treatment options are becoming available over time [3,4]. HIV can be classified as Type 1 or Type 2, with HIV-2 being less infectious and uncommon [5]. HIV-1 is generally prevalent in East Africa while HIV-2 is found in West Africa but epidemics exist in India, Brazil, Portugal, and Guinea-Bissau, among other countries [5,6,7,8]. In the United States, adolescents, African American men, and young adults (13–19 years) have higher incidences of HIV infection, according to past data [9,10,11]. Health disparities and gender play a role in HIV analysis because they are linked [12,13]. Removing healthcare barriers and providing primary and secondary AIDS prevention can reduce the disparity in care [14]. Additionally, the correlation between poverty and HIV incidence has shown conflicting data to determine a true linkage [15,16].

Advances have not only been made in treatment but also in HIV diagnostics. The detection of HIV was conventionally performed through lab-intensive examinations but has progressed to modern-day “lab on a chip” modalities. Streamlining the processes of detection offers a convenient method for on-the-go HIV recognition for early-stage disease identification [17]. The current gold standards for diagnosis and monitoring include flow cytometry, PCR, and ELISA. However, these techniques are expensive, time-consuming, and require skilled technicians to use them. The standards set by the World Health Organization (WHO) characterize point-of-care testing as affordable, sensitive, specific, user-friendly, rapid and robust, equipment-free and deliverable, or ASSURED [18]. By following the WHO’s guidelines for point-of-care testing, novel and innovative microfluidic devices can bridge the gap in testing and managing HIV in resource-limited areas. Evaluation of an HIV prognosis requires complex lab infrastructure and high cost for viral load testing, which may not be readily available in resource-limited settings [19]. This necessitates the need for faster, sensitive, and easily transportable microdevices that can ensure available testing. Advances in technology permit the production of handheld devices and microfluidics, including lensless imaging with smartphones and multiplexing [18]. Furthermore, CD4+ T cell counting, sample preparation, and nucleic acid molecular diagnostics, as a result, have been enhanced with the production of these new microfluidic devices [17]. As point-of-care devices become readily available, high-throughput methods can speed up population testing; however, this may come as a tradeoff to portability or processing time [18,20]. The capabilities of these tests can extend to HIV screening, load monitoring, and infant diagnosis [18]. To compete with the currently used conventional methods, such as flow cytometry, PCR, and ELISA, these devices have to be reliable and effective as well as inexpensive to be used in resource-limited settings. The aim of this review paper was to explore the current methods and trends involving “lab-on-a-chip” microfluidic devices for HIV detection and monitoring in point-of-care (POC) settings.

## 2. HIV, CD4+ T Cells, and AIDs

HIV virions are approximately 100 nm in diameter and are encased in an envelope with surface glycoproteins. The interior capsid surrounds two identical single-stranded RNA strands [5]. Once the virus attaches and fuses with the CD4+ T cells, the reverse transcriptase generates viral dsDNA. Integrase fuses the HIV genome to the CD4+ T cell’s DNA-permitting host transcription. As more HIV proteins are produced and assemble into functional HIV particles, they are released from the CD4+ T cell through budding [21]. This repeated cycle causes a continuous decrease in CD4+ T cells through a caspase-1-mediated pyroptosis or caspase-3 activation [22,23]. Furthermore, the immune system is activated from HIV gene products Vpu, Nef, and Tat, which allow the virus to increase replication [24,25]. Late-state progression of HIV leads to AIDS due to the compromised immune system. Typically, AIDS is defined as less than 200 cells/µL for CD4+ T cells [26]. This increases the risk for infections to arise, due to the immunocompromising effects of HIV. Antiretroviral treatment can assist in the prevention of AIDS but monitoring CD4+ T cell count and viral load through testing and point-of-care services can help in disease management [27]. The primary tests for AIDS detection are inclusive to HIV detection, such as ELISA and viral load tests. These diagnostic tests serve to detect HIV markers, such as reverse transcriptase, gp120, or p24, or by amplifying HIV DNA to detectable levels. Alternatively, analytes can include HIV antibodies, such as anti-gp41 or anti-gp120. Currently, there is no cure for HIV and treatment options rely on suppressing viral replication with the use of highly active antiretroviral therapy (HAART) [28,29]. After the discontinuance of HAART, there tends to be a plasma viral rebound of long-term latent cells [30,31,32]. Patients have to be monitored to prevent potential resistance to the antivirals administered, which can result in drug-resistance and treatment failure. Successful inhibition is denoted by a viral load under 200 copies of viral RNA per milliliter of blood whereas, if the viral load is greater than 1000 copies/mL after six months of treatment, it may indicate treatment failure [33,34,35]. While conventional detection devices are expensive, microfluidic devices can be constructed for on-the-go analysis of CD4+ T cells, HIV RNA, or DNA levels to monitor disease progression in resource-limited areas.

## 3. Sample Preparation: A Major Challenge for HIV Testing

A major issue in POC HIV diagnostic devices is the lengthy sample preparation time that varies across various technologies and assays. Microfluidic devices for rapid HIV detection should have minimal sample preparation to be efficient and should provide rapid results. Unfortunately, current devices involve extensive sample preparation steps that prolong the overall time it takes to achieve diagnostic results. Traditional benchtop methods, such as PCR and ELISA, require expertise and extensive sample preparation but scalability to microfluidic devices offers rapid quantification of viral proteins and detectable loads to provide on-site results. Conventional off-chip sample preparation involves the preparation, mixing, washing, and buffering of reagents, which can take up to a few hours. Benchtop PCR, for example, requires the generation of cDNA and primer mixing, which alone can take 1 to 3 h. Automated detection systems are able to rapidly quantify virions and pre-load the microfluidic devices with the necessary reagents in a pre-packaged format. This has been implemented for zika virus detection, with detection times up to 40 min. The on-heating chip capability, temperature control, and reagent pre-loading provide the means to rapidly quantify viral loads in patient samples with zika virus [36]. The incorporation of these systems into HIV diagnostic tools have been shown to provide rapid results, from 60 to 90 min, while providing accurate results [37,38,39]. Novel HIV diagnostic technology, such as paper- and flexible material-based assays, provide a variety of designs with a simple fabrication process and the capability to be mass produced. Paper-based detection methods have been shown to decrease the detection time by reducing the sample preparation time and consolidating the entire assay process to take up to 60 min [40,41]. However, the variations in the specificity and sensitivity in these devices limits the functional use of these devices for HIV detection [42].

## 4. Microfluidic Devices for Point-of-Care Applications

Microfluidic devices are small-scale chip-based devices that contain miniaturized channels and chambers to facilitate chemical reactions. Physical forces, such as electrokinetics and capillary action, are able to mix the samples on-chip. Often times, low volumes of reagents in the microliter range are used for detection [43]. Miniaturizing laboratory techniques into an on-chip reaction can be a tedious process that places limitations on material fabrication and costs, reagent volumes, and scalability of reactions [44]. Therefore, novel methods to fabricate affordable microfluidic devices are imperative because they can bypass the inherent limitations in providing devices that can rapidly assess HIV status with great accuracy. Unfortunately, not all chip materials or fabrication processes are streamlined or cost-effective. In this section, we describe the challenges, limitations, and advantages of various diagnostic assays with a focus on affordable microfluidic devices utilized for HIV detection in resource-limited settings. Table 1 provides a comparison between different microfluidic devices and diagnostic assays used for HIV detection.

### 4.1. Polymerase Chain Reaction (PCR)-Based Devices

PCR is a technique that requires a thermocycler due to each stage requiring different temperatures. This method exponentially increases the nucleic acid’s quantity in each cycle. This is represented as 2^n^, where n is the number of cycles. The three stages are denaturing, annealing, and extending. Denaturing separates the DNA strands on which primers attach during the annealing stage. The strands are extended during the final stage, amplifying the DNA. The requirement of a thermocycler provides a hurdle for resource-limited areas because it is expensive and requires a trained technician. Another method, known as quantitative PCR (also known as real-time PCR), utilizes fluorophores to produce real-time readings. The optical filters used are expensive and offer a bottleneck in cost for resource-limited areas [45]. Reverse transcription PCR (RT-PCR) and qPCR can be used to measure gene expression by synthesizing cDNA from mRNA. In the case of HIV, viral mRNA can be detected by these machines for analysis of viral load testing. Root et al. utilized a polymer capture matrix to purify serum DNA and RNA, specifically the gag region of HIV. The four-chamber microfluidic device was composed of polymethylmethacrylate with a bottom substrate and middle and top layers combined through solvent bonding. The capture matrix was composed of polyacrylamide through free-radical polymerization. While this device can purify RNA from a large sample volume, additional PCR steps have to be taken off-site for data collection [46]. Lee et al. fabricated a two-layer cyclic olefin copolymer microfluidic chip with five chambers for RT-PCR for HIV detection. The top layer contained grooves for silicone tubes containing microvalves to control the inputted fluid. The bottom layer was fabricated with micropatterned nickel. The developed RT-PCR microfluidic chip was able to detect HIV p14 and gp120 in less than an hour [47]. PCR-based microfluidic devices are generally not disposable due to the high cost of materials. Lower fabrication costs can be attributed to new materials used, such as polydimethylsiloxane, polymethylmethacrylate, and polycarbonate, to create microfluidic chambers [48,49,50]. Miniaturization of PCR to a handheld device still requires changes to be made. Conventional PCR devices can have a poor reaction efficiency, potential for false negatives, and a slow thermal transition speed [51]. Compared to conventional PCR, a point-of-care PCR device is capable of faster thermal cycling due to rapid heat transfer in the small reactor as well as portability with small reactant quantities [52]. Jangam et al. fabricated an assay card capable of PCR reagent storage, thermocycling, fluorescent detection, and PCR mix assembly with a portable analyzer to detect HIV infection in infants. The assay card is composed of polypropylene and contains input ports for reagents and an exit channel for venting. Fluorescence can be monitored through the optical window on the chamber edge. Additionally, the analyzer performs mix assembly and quantitative PCR once the assay card is placed and disposed. The cost of the analyzer is less than $3000 compared to commercial instruments ranging from $17,000 to $25,000 [53,54]. With respect to PCR, price is a major constraint as laboratories may not have the proper infrastructure or trained personnel to perform the experiments. As affordable materials are made more readily available, PCR can replace the need for complex infrastructure. The assay card is cost-effective since it is composed of polypropylene with injection molding and does not involve time-consuming processes, such as photolithography, leading the price to be $50/assay card [54]. While PCR is regarded as a gold standard for infant HIV screening, further investigation and studies are needed to devise potent point-of-care devices for rural areas that can emulate the sensitivity and specificity of traditional benchtop PCR.

### 4.2. Isothermal Amplification-Based Devices

#### 4.2.1. Loop-Mediated Isothermal Amplification Devices

**Figure 1:**
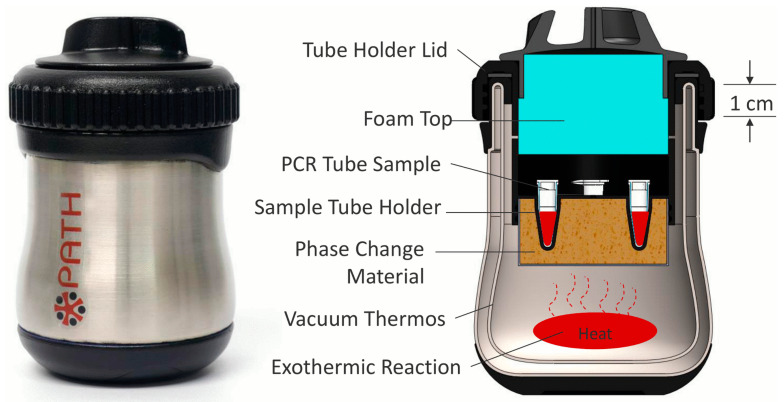
Cross-section showing the internal components of the LAMP-based device. Approximate dimensions of the heater are 80 mm in diameter by 120 mm in height. Reprinted with permission from ref. [61].

Loop-mediated isothermal amplification (LAMP) is a molecular test for nucleic acid amplification as an inexpensive substitute for PCR. Compared to the temperature fluctuations in PCR, LAMP utilizes a constant temperature between 60 and 70 °C. LAMP uses 4 to 6 primers that are extended by DNA polymerase for molecular amplification, with a similar principle to PCR [55]. However, LAMP requires no thermal cycling and is a highly specific, fast, and portable diagnostic test for infectious diseases [56,57]. Recently, RT-LAMP has been extensively used for COVID-19 testing and provides applications in HIV detection as well [58,59]. This favorable nucleic acid amplification test (NAAT) is preferred over PCR for field use and is capable of real-time detection and measuring fluorescent intensity [60]. Electricity-free RT-LAMP devices have been previously constructed, powered by an alternative source of energy. In order to overcome the hurdle of electricity availability in resource-limited countries, Singleton et al. devised an electricity-free non-instrumented nucleic acid amplification (NINA) device paired with a nucleic acid lateral flow (NALF) platform. The heating system involves an exothermic reaction from magnesium oxidation, which transfers heat to the phase change material, palmitic acid (Figure 1). Biplex detection of HIV and β-actin, an internal control, was performed from normal human plasma; however, the inclusion of β-actin primers decreased HIV-1 detection sensitivity. Despite this, HIV detection was reliable and performed under 80 min with the NINA-paired NALF device for RT-LAMP [61]. Curtis et al. assessed a non-instrumented nucleic acid amplification single-use disposable (NINA-SUD) RT-LAMP point-of-care device with heat generated from an exothermic reaction for HIV detection [62]. Similar to Singleton et al., the exothermic reaction was initiated by the addition of saline from an external reservoir, which reaches a magnesium iron fuel powder packet that permits heat output. Measures to increase holdover time and reduce heat loss involved the fitting of polyvinyl chloride foam as an insulation, attached with acrylic tape, and the addition of graphene nanoparticles with palmitic acid to increase the thermal conductivity of the material. Furthermore, palmitic acid was thermally coupled with the Mg-Fe to act as a phase change material. The results indicate comparable to superior performance of whole blood HIV analysis compared to thermal cycling with the NINA-SUD, which can be used as a rapid diagnostic test [62]. A separate pH-based RT-LAMP assay for HIV detection was produced using a metal-oxide semiconductor with rapid detection speed [63]. An electrical signal is produced by ion-sensitive field effect transistors in the microfluidic chambers when pH changes due to hydrogen production. Sensitivity of this device was lower on-chip (88.8%) compared to in vivo (95%), likely due to the low reaction volume [63]. These methods possess limitations, such as multiple processing steps, lower sensitivity, and off-chip sample preparation. Sample preparation, in particular, can be crude because LAMP is less affected by preparation compared to PCR [64,65]. Additionally, LAMP is more cost-effective compared to PCR, as Nliwasa et al. purchased LAMP tests in a batch of 14 samples for $9.98 [66]. However, the purchasing cost of LAMP tests can vary from $13.78 to $28.34 [67]. However, LAMP can also use fluorophores in its detection mechanism, which can raise the cost. Despite these shortcomings, these methods are capable alternatives to traditional PCR in resource-limited areas as these devices are portable, inexpensive, provide fast results, and utilize alternative power sources where electricity may not be readily available.

#### 4.2.2. Recombinase Polymerase Amplification

**Figure 2:**
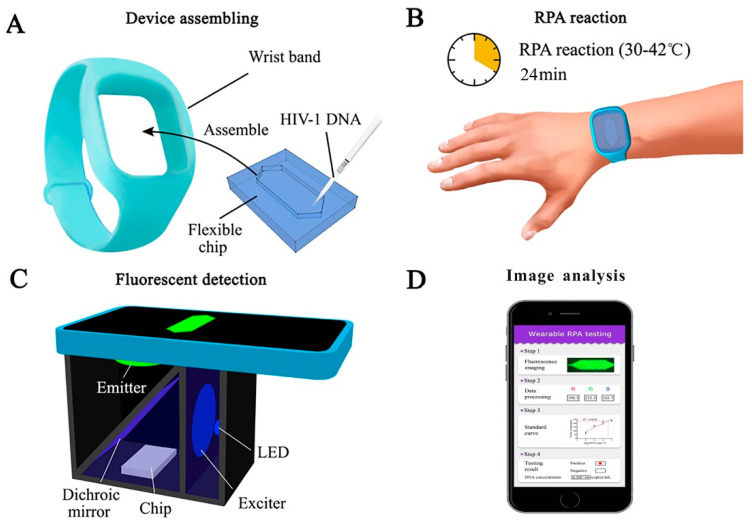
Schematic of a wearable RPA device for HIV diagnosis. (**A**) A wearable and flexible chip made of PDMS. (**B**) RPA reagents and HIV-1 DNA assembled in the wristband and processed by human body heat. (**C**) A cellphone-based fluorescence detection system used to record the amplification results. (**D**) Image analysis conducted through ImageJ to quantify the fluorescent signals. Reprinted with permission from ref. [79]. Copyright 2022, Elsevier.

Recombinase polymerase amplification (RPA) assay is a form of isothermal amplification. It uses fewer primers compared to LAMP and can be combined with a fluorescent probe [68]. The underlying mechanism behind RPA involves the use of a recombinase protein to bind primers, forming a complex. This complex scans for homologous sequences in DNA and then the primers are inserted by the recombinase. Afterwards, the recombinase is deconstructed, and DNA polymerase is able to elongate the primers [69]. Similar to LAMP, no thermal cycler is required, which makes this method reliable over PCR for portability, speed, and cost-effectiveness [70]. There is minimal sample preparation and RPA is rapid due to exponential DNA replication, which can produce results within 20 min for HIV and other viruses [71,72,73]. RPA can be used with reverse transcriptase, known as RT-RPA, to detect RNA molecules by synthesizing the corresponding cDNA [69]. Additionally, RPA is cost-effective compared to PCR as costs can go as low as $4.45 [74]. One study used RPA-detected HIV DNA in the temperature range of 25 to 42 °C in infants. Immunochromatographic strips (ICS) were used for endpoint detection with the RPA assay and demonstrated success due to its sensitivity. The sensitivity of the device allowed the detection of HIV-1 from a blood droplet with amplification of less than 10 copies of HIV proviral DNA. The long terminal repeat (LTR) primer and pol primer used were able to amplify the proviral DNA with success on multiple HIV-1 subtypes. The major limitation of using ICS with RPA is the risk of cross-contamination because the reaction tubes must be opened to add the mixture to the ICS. Although a device that bypasses this step has been devised, contamination poses a risk for sample purity with untrained individuals that use point-of-care devices [71,75]. RT-RPA is capable of detecting all the major subtypes of HIV-1 groups M and O as performed by Lillis et al. The results indicate RNA sensitivity is 98.1% and DNA is 97.2%, with the overall sensitivity of the assay performing at 97.7%. Moreover, no false positives were detected when tested with alternate genomes, such as simian immunodeficiency virus (SIV) [76]. The reagents used in RPA can be stored for up to 3 months at 25 °C, while they last up to 3 weeks at 45 °C. This proved to have no impact on RPA performance but storage for longer than 3 weeks at 45 °C reduces assay sensitivity. No cold chain storage is necessary and the assay performs well even if the reagents are briefly exposed to 45 °C [77]. Real-time RPA (qRPA) is combined with a fluorescent probe to detect HIV-1 DNA levels. One study provided the proof-of-concept of quantitative RPA with an internal control where the fluorescent data were analyzed by a MATLAB script. The qRPA assay was able to detect viral DNA 100% of the time with sensitivity varying across sample concentrations, providing evidence that qRPA can be used for point-of-care settings with inexpensive fluorescence readers. A drawback of this method is the precise control of amplification because RPA lacks true PCR cycles. The precise control of temperature allows for the modulation of RPA cycles to prevent thermal degradation and to control DNA amplification as well as maintain reagent consistency to prevent errors. Moreover, there is the risk of the probes becoming photobleached but provided the right components, this can be prevented [33]. As technology improves, new devices come to light, such as wearable devices. Wearable platforms can provide portability and allow wireless connection to smartphones for health monitoring [78]. A wearable RPA device was created by Kong et al., which is an efficient method compared to traditional nucleic acid amplification techniques. The RPA device was fabricated with polydimethylsiloxane (PDMS) with 50 µL RPA reagent volumes for testing (Figure 2). The device used human body heat to amplify HIV-1 DNA in under 30 min. This method is advantageous as it provides an alternative to electric power and uses body heat instead. The rapid testing, portability, and alternative power source provided by this wearable device makes it favorable for use in resource-limited areas since it can serve to detect and manage HIV-1 [79]. RPA in its many forms is a powerful tool for diagnostics in resource-limited settings and should be further explored with variations to improve cost-effectiveness and sensitivity.

### 4.3. ELISA

**Figure 3:**
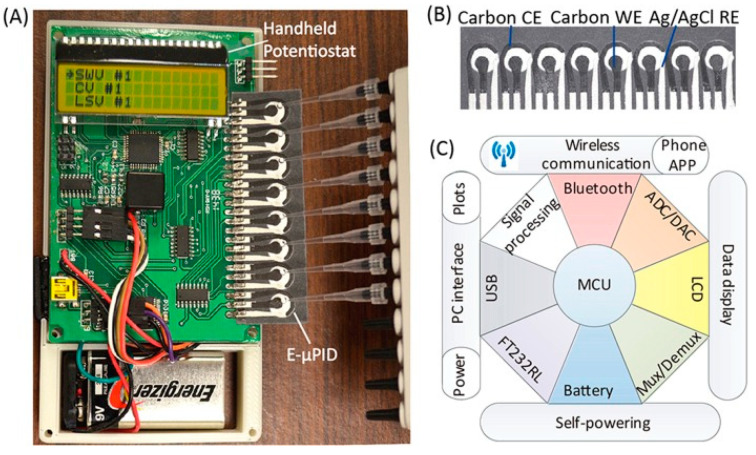
A portable paper-based diagnostic platform integrating an electrochemical microfluidic paper-based immunosensor array (E-µPIA) and a handheld potentiostat. (**A**) The handheld potentiostat inserted with an E-µPIA. (**B**) The E-µPIA. (**C**) Schematic architecture of the potentiostat circuit constructed based on a microcontroller unit (MCU). Reprinted with permission from ref. [99]. Copyright 2022, AIP Publishing LLC.

Enzyme-linked immunosorbent assays (ELISA) or enzyme immunoassays (EIA) operates on the principle of antigen or antibody binding to identify molecular interactions. Antibodies are proteins produced by immune cells, such as plasma cells, in response to an infection. Pathogens express antigens on their surface, which can bind to an antibody, resulting in the formation of an antigen–antibody complex. These antigen–antibody complexes can elicit the termination of pathogens through neutralization, agglutination, precipitation, or opsonization (complement fixation). ELISA has varying methodologies depending on the method used. Direct, indirect, sandwich, and competitive ELISA all use antibodies to bind to antigens but vary in the order of binding. The substrates used are most commonly horse radish peroxidase or alkaline phosphatase because they generate a color change in the assay [80]. Early detection of HIV can be completed with ELISA with a follow-up Western Blot for a confirmatory diagnosis [81]. An indirect ELISA is normally conducted for HIV detection from a blood or saliva sample and has a greater sensitivity than direct ELISA [80]. HIV antigen p24 can be detected through ELISA during the onset of a symptomatic primary infection [82]. The currently used tests are fourth-generation assays, which are highly sensitive and specific with 100% sensitivity and 99.5% specificity [83,84,85]. Fourth-generation assays are able to detect the p24 antigen while reducing the amounts of present false negatives and false positives. Developing a point-of-care diagnostic device with ELISA can permit highly sensitive and specific detection of diseases in resource-limited settings. However, a major drawback of ELISA testing involves a long wait time, often 6 to 8 h, to obtain results [80]. Various microfluidic ELISA devices have been made with variations, such as the implementation of glass capillaries or removing an enzyme label [86,87]. One ELISA innovation is known as the mChip assay, which was tested in Rwanda. The mChip is cost-effective, portable, and can diagnose both HIV and syphilis. Comparable to benchtop methods, the HIV detection sensitivity and specificity are 100% and 96%, respectively, with this device [88]. A major benefit of this device is the rapid delivery of results in under 20 min without the need for a trained individual to interpret results [88]. Other devices, such as lateral flow assays used in HIV detection, require personnel to execute the test and interpret the results, making it susceptible to errors, such as false positive readings [89,90,91]. Apart from using point-of-care devices to detect and amplify HIV strains, levels of CD4+ cells can be detected and counted instead. A microfluidic ELISA developed by Wang et al. performs rapid counting of CD4+ cells by capturing them in microfluidic channels with antibody-functionalized magnetic beads [92]. Analysis is performed by a smartphone application, which rapidly reports the results under 10 min. While this test is currently not used in HIV detection, it can be implemented for point-of-care AIDS management by assessing blood levels of CD4+ cells for antiretroviral therapy [92]. Modalities for rapid quantification have been demonstrated to provide robust specificity and efficiency, particularly with ImageJ algorithms and the use of magnetic beads for CD4+ cell counting [93,94]. A cost-effective platform can meet the need for rapid T lymphocyte detection in AIDS management [27]. Alternatively, in areas that lack resources to manufacture immune assays, paper- and flexible material-based assays can be used. Paper and flexible materials are affordable, disposable and can be mass produced. Known as P-ELISA, it can be fabricated through photolithography and can detect HIV antigen gp41 with colorimetry. Further methods of paper microfluidic fabrication include inkjet printing, wax printing, or plasma treatment [95,96,97]. The targeted antigen and their antibodies are immobilized on the P-ELISA followed by the blocking of non-specific binding. The unbound proteins are then washed out and an indirect P-ELISA is performed. This method is less sensitive than ELISA but it can be completed in under an hour, small reagent volumes are used, and simple equipment is needed [98]. Another study used paper-based ELISA to detect and diagnose HIV and HCV co-infections. The device is advantageous with multiplexing and has high sensitivity. Samples were detected against HIV p24 and HCV core antigens with the use of eight electrochemical immunosensors (Figure 3) [99]. While multiple studies have tested paper-based devices, these devices should be translated into regular production and commercialized for low-resource areas [100,101]. Developing paper technology can provide a benefit to areas that are constrained with resources as a new method for constructing ELISA, for which plate readers can cost up to $20,000 [102]. The primary limitations faced by paper-based devices include reagent exposure to harsh conditions during transport and varying degrees of specificity and sensitivity between devices [103]. Further research should be applied in paper and flexible microdevices to foster improvements as they can serve as potent tools in HIV detection with commercialization.

### 4.4. ELISA Alternatives

**Figure 4:**
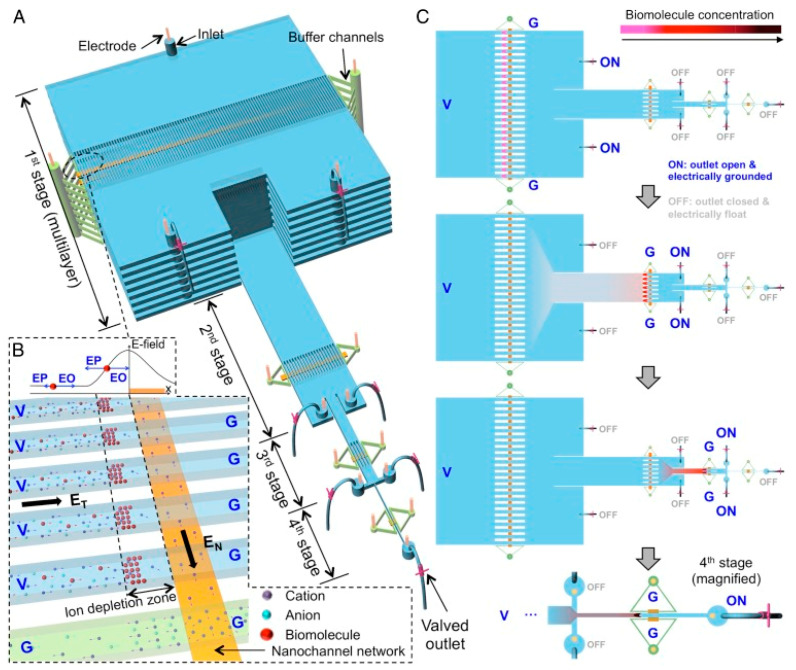
Principle of HOLMES. (**A**) Schematic of HOLMES with hierarchical multistages. At each stage, parallel microchannels and buffer channels are bridged by a thin nanochannel network patterned on the bottom of the microchannels. (**B**) Schematic of nanofluidic biomolecule concentration in massively parallel channels. Under the electrical configuration shown, biomolecules are electro-osmotically injected into the parallel channels and electrokinetically concentrated in the ion depletion zones induced near the micro-nanochannel junctions. (**C**) Schematic of relayed reconcentration of biomolecules from massively parallel microchannels into a single microchannel to dramatically boost the concentration performance. Reprinted with permission from ref. [105].

While benchtop methods, such as PCR and ELISA, are considered gold standards for molecular detection and protein quantification, some new microfluidic devices have been put to the test [104,105,106]. A novel device known as hierarchical nanofluidic molecular enrichment system (HOLMES) consists of a series of microchannels between different stages (Figure 4). The first stage has the microchannels stacked vertically but as the stage progresses, the number of microchannels decreases. The final stage has one microchannel. This device operates through electro-osmosis and gravitational flow. Biomolecules are concentrated at the first stage but are then transferred to the second stage and then reconcentrated. This process repeats until the biomolecules reach the final stage, improving the concentration performance [107]. This method is capable of detecting nucleic acid and proteins at low concentrations in human sera. HOLMES is capable of detecting HIV p24 proteins with concentrations as low as 10 aM within 60 min [105]. ELISA has a detection limit around 1 pM with a longer time to obtain data, from within hours to a day [108]. Another highly sensitive automated device implemented by Hughes and Herr, which is capable of multiplexing, can detect HIV within 60 min. Known as µWestern blot, it is a rapid quantitative assay of a traditional Western blot but miniaturized for microfluidic use [109]. Western blots are a method used to detect proteins based on their molecular weight through gel electrophoreses. Molecular weight-based separation can be utilized for HIV detection as HIV proteins have different molecular weights. Traditional Western blotting is resource-intensive, requires primary antibodies, and has difficulty with data throughput. This makes Western blotting unfavorable for areas that are resource-limited since antibody purchases can be expensive for some proteins [110]. Additionally, a confirmatory HIV diagnosis with traditional Western blotting involves the reactivity of two or more of the gp120/160, p24, and p41 bands being as intense as a p24 band with a weak control serum [111]. Hughes and Herr’s device was tested on multiple markers for HIV, such as reverse transcriptase, gp120, and p24, with red fluorescent primary antibodies. The µWestern blot had a low limit of detection (10 aM) and was able to detect HIV proteins in weakly reactive human sera. Additionally this device confers a 10^3^ reduction in reagent quantity (starting sample volume of 2 µL), fast run time (10–60 min), and multiplexing, making it beneficial for use in rapid HIV detection [109]. Sia et al. developed a portable and cost-effective immunoassay, which operates on the principle of silver reduction and optical detection to shorten assay time. Termed POCKET immunoassay, it is fabricated with PDMS and detects anti-HIV-1 antibodies. While microfluidic ELISA poses problems with detection under continuous flow and poor sensitivity under optical detection, POCKET immunoassay uses antibodies conjugated to gold colloids, which catalyze silver reduction [106,112]. The silver film produced by the reduction in the number of silver ions is a function of the marker to be detected, in this case, anti-gp41. This method can quantify the amount of anti-gp41 in sera and differentiate between infected and non-infected HIV-1 patients. The benefits of this device include signal amplification, no photobleaching, a low price point, portability, battery power, and long-term stability and use. This makes it beneficial for use in areas that are limited in supplies because it is cheap and reusable [106]. ELISA alternatives, such as HOLMES, POCKET immunoassay, and µWestern blotting, serve as different routes for disease detection in resource-limited areas. These alternatives are cost-effective, rapid, and highly sensitive, serving as point-of-care devices in place of traditional ELISA, which may not be readily available.

**Table 1:** A comparison of microfluidic HIV tests.

Method	Advantages	Disadvantages	References
LAMP/RT-LAMP	No thermal cycler needed, isothermal cycling, inexpensive ($9.98–$28.34), highly specific, alternate power sources (magnesium oxidation), rapid testing, 4–6 primers used	Lower sensitivity than qPCR, multiple steps for preparation, potential contamination as sample is prepared off-chip, difficulty in multiplexing	[64,65,113]
PCR/RT-PCR	Highly sensitive (>99%) and specific (>98%), can include fluorophores, fast thermal cycling	Thermal cyclers are expensive ($17,000–$25,000), requires a trained technician for benchtop apparatus, off-chip sample preparation, long wait time (hours–days)	[47,52,53,54]
RPA/RT-RPA	No thermal cycler needed, can include fluorophores, minimal sample preparation, rapid, highly sensitive (97.7%) and specific, reagents can be stored for up to 3 months at 25 °C, 2–3 primers used, affordable ($4.45)	Precise control of amplification is necessary, risk of photobleaching of probes, risk of contamination with ICS-RPA	[33,71,75,76,77]
ELISA	Highly sensitive (100%) and specific (>99.5%), portable, reliable results, can include fluorescent conjugates, can be utilized in cell counting	Benchtop has a long wait time (days) to obtain results, antibodies can be expensive to purchase	[80,83,84,85,88]
P-ELISA	Affordable, small reagent volumes, simple equipment, results in under an hour, capable of multiplexing	Less sensitive (10× lower) than normal ELISA, sensitivity and specificity vary between devices, reagents can be evaporated during transport	[98,99,103]
HOLMES	Detect HIV p24 as low as 10 aM, rapid, works on proteins and nucleic acids	Antibodies can be expensive to purchase, fluorescent microscope needed if using fluorescent labels	[105]
µWestern Blot	Rapid, include fluorescent antibodies, multiplexing, small reagent volumes	Antibodies can be expensive to purchase, smaller pore size can cause large antibody probes to be immobilized irreversibly	[109]
POCKET Immunoassay	Sensitive, affordable, antibodies conjugated to gold catalyze the reaction, reusable	Manual pipetting into the microwells poses a risk for contamination	[106]
Smartphone-based Detection	Replaces need for thermocycler or microscopes, can interface with microfluidic chips, affordable assays, powerful imaging, data analysis through applications, highly sensitive	Privacy and security concerns when storing medical data on applications, manual functionalization can result in chip-to-chip variability, manual pipetting has a risk for contamination	[114,115,116,117]

## 5. Smartphone-Based Devices

**Figure 5:**
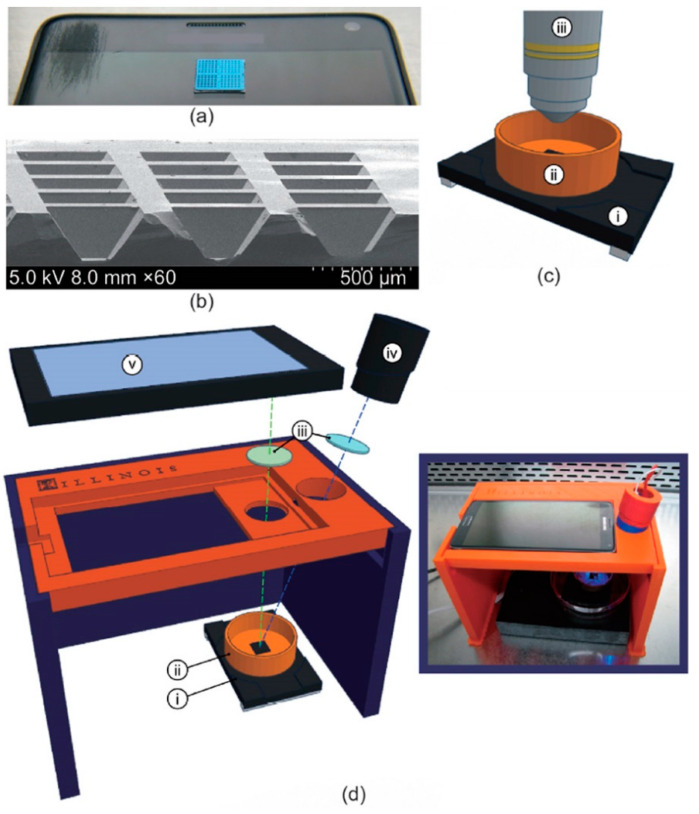
(**a**) Image of 1 cm × 1 cm silicon microchip substrate sitting on a Samsung smartphone. (**b**) Scanning electron microscopy cross-section of 160 µm deep reaction wells. (**c**) Schematic of microchip and heating stage in fluorescence microscope apparatus, including: (i) heating stage, (ii) copper base containing mineral oil, and (iii) fluorescence microscope objective. (**d**) Expanded diagram of smartphone LAMP apparatus, including: (i) heating stage, (ii) copper base containing mineral oil, (iii) wavelength filters placed in front of the LED and smartphone camera, (iv) blue LED light source, and (v) smartphone. (d-inset) Image of apparatus assembled in biosafety cabinet. Reprinted with permissions from ref. [115].

Smartphones and cellular devices are commonly used devices throughout the world, both in developed and developing countries. Smartphones are user-friendly and most provide an interactable touch interface. Most individuals in developed countries have access to or own a smartphone, as indicated by 81% of Americans having ownership of smartphones in 2019 [118]. Even in developing countries, an increasing number of individuals have access to a smartphone. According to a 2017 survey, 51% of individuals in South Africa have access to a smartphone, while 91% have access to a cellular device. Additionally, smartphone ownership has been increasing over the years, indicating greater access to cellular devices in rural or underserved areas [119]. With the multivalent capabilities of smartphones, they can be used in point-of-care settings for disease detection. Some systems have been developed using smartphones for microscopy, colorimetry analysis, and genetic testing [92,120,121,122]. By incorporating smartphones into HIV detection, improved patient monitoring, epidemiological tracking, and early-stage diagnoses can be facilitated. Current smartphone-based devices have been used to diagnose and detect infectious diseases, including HIV [92,123,124,125,126]. One study performed RT-LAMP with HIV-1 and used a smartphone for fluorescence imaging for viral load interpretation (Figure 5). The incorporation of a smartphone replaces the need for detection equipment, such as a fluorescence microscope [115]. This is cost-effective as this equipment is expensive and may not be readily available in resource-limited areas [70,127]. Furthermore, traditional laboratory assays, such as ELISA, can be developed on a chip and then imaged through a smartphone. Chen et al. created a platform which interfaces with a mobile device to conduct ELISA [116]. The smartphone triggers the device to supply energy to a printed circuit board that can hold the microfluidic chip for ELISA. Imaging can be conducted through the smartphone and then transmitted to a computer for processing or analysis can be conducted on the smartphone with programmable applications [116]. A study published in 2017 used microelectromechanical piezoelectric surface acoustic wave (SAW) sensors to diagnose HIV with smartphones. This highly sensitive proof-of-concept study detected HIV within seconds by speeding up the diagnostic process through the use of smartphones. This method is equipment-free, is affordable by cutting out expensive equipment, and reduces the risk of false positives by multiplexing arrays of biochips. The sensing area is composed of functionalized quartz used to capture the targeted proteins, such as p24. The limit of detection and lowest detected concentration associated with this device are 1.1 nM and 2 nM for anti-p24 antibodies, respectively. Furthermore, the speed at which results are delivered are very rapid because HIV antibody concentrations can be interpreted in 10 s after sample insertion. The limitations associated with this study include manual functionalization, which can cause variability from biochip to biochip, and manual pipetting for sample insertion, which poses a risk for contamination [114]. Innovative devices using smartphones, such as this one, can meet the WHO’s ASSURED criteria for point-of-care services in underserved areas. In another experiment, Gray et al. used SAW biosensors with a smartphone-connected prototype reader for a digital readout for HIV detection from clinical samples. The samples were electronically interpreted very rapidly, with results provided in under a minute. The dual-channel biochips used were functionalized for HIV proteins gp41 and p24 detection. Functionalization was conducted by inkjet printing, which kept variability between biochips low. Once tested, the assay demonstrated 100% sensitivity for anti-gp41 detection and 100% overall specificity. However, the anti-p21 biomarker sensitivity was 66.1% [128]. A caveat associated with smartphone-based technology is related to cybersecurity. Storing sensitive medical data on a smartphone application can lead to privacy and security concerns [117]. Future applications should include device security to prevent data theft and ransomware [129]. Overall, smartphone technology has advanced greatly since their initial conception. The implementation of smartphones for HIV detection in underserved areas can help increase testing while also serving functions in AIDS management. Compared to standard benchtop assays and devices, such as PCR and ELISA, smartphone usage is widespread and it offers no shortage of these devices for a cheaper cost. Future applications for HIV detection should focus on developing smartphones as a method of detection since they have powerful processing power capable of rapidly analyzing data.

## 6. Conclusions

The implementation of point-of-care devices for HIV diagnostics in resource-limited settings can serve to bridge the need for increased testing and a lack of resources. Development of new technologies, including smartphones, offers a wide modality of assays that can be used for disease detection. Additionally, paper and flexible assays provide an avenue for inexpensive materials to be used for assays, which minimizes the cost of purchasing or developing expensive equipment. Traditional laboratory methods are resource-intensive and some require trained personal to interpret the results compared to point-of-care devices. Miniaturizing benchtop machines translates their functionality to a microscale for rapid analysis. Microfluidic point-of-care devices are also highly sensitive and specific, similar to their benchtop counterparts. They are also inexpensive due to requiring low-cost materials for fabrication and can be reusable or disposable. Rapid HIV testing can be accomplished with these various technologies but the option of which method is ideal differs based on the needs and environmental or financial restrictions. Resource-limited areas may lack complex laboratory infrastructure, which can make point-of-care devices preferable for HIV detection. Cost-effective methods stray away from PCR as thermocyclers are expensive; therefore, LAMP or RPA can be used instead because isothermal methods do not require the use of a thermocycler. Moreover, future applications of microdevices for diagnostics will head in the direction of smartphone applications. Modern smartphones have a powerful processing capability and are able to take images in a wide wavelength of light. This permits smartphones to be used as a power source for microfluidic devices or host applications that can analyze data. In resource-limited regions, emphasis should be placed on using RPA or LAMP compared to tedious methods, such as PCR or ELISA, for HIV diagnosis. Emerging technologies, such as smartphone-based detection, paper-based methods, and novel fabrication methods, should be tested in these regions to evaluate their efficacy for HIV detection. In areas that are not constrained by infrastructure or resources, PCR or ELISA should be used as the optimal standards for HIV detection. The future direction for HIV diagnosis and monitoring needs to place an emphasis on low-cost materials with rapid results and low wait times. Conventional detection methods are costly and often take a long time (hours to days) to achieve reportable results. Resource-limited and underserved regions are unable to have the proper infrastructure and equipment for accurate diagnosis and monitoring, developing the need for low-cost diagnostic systems. However, this is limited by the fabrication complexity, material costs, and accuracy. By developing novel microfluidic diagnostic assays that are affordable and accurate, it is possible to overcome this barrier to develop novel POC devices for resource-limited regions. Additionally, the transition of microfluidic devices from laboratory development to field testing requires their commercialization for widespread impact. Hence, further research should be focused on developing novel microdevices that are able to be mass produced because they can be implemented for detecting HIV at an early stage for immediate treatment and management.

## Data Availability

The data presented in this study are available on request from the corresponding author.
